# Cellular RNA Helicase DDX1 Is Involved in Transmissible Gastroenteritis Virus nsp14-Induced Interferon-Beta Production

**DOI:** 10.3389/fimmu.2017.00940

**Published:** 2017-08-09

**Authors:** Yanrong Zhou, Wei Wu, Lilan Xie, Dang Wang, Qiyun Ke, Zhenzhen Hou, Xiaoli Wu, Ying Fang, Huanchun Chen, Shaobo Xiao, Liurong Fang

**Affiliations:** ^1^State Key Laboratory of Agricultural Microbiology, College of Veterinary Medicine, Huazhong Agricultural University, Wuhan, China; ^2^Key Laboratory of Preventive Veterinary Medicine in Hubei Province, The Cooperative Innovation Center for Sustainable Pig Production, Wuhan, China; ^3^College of Life Science and Technology, Wuhan Institute of Bioengineering, Wuhan, China; ^4^College of Life Sciences, South-Central University for Nationalities, Wuhan, China

**Keywords:** transmissible gastroenteritis virus, non-structural protein 14, DDX1, interferon-beta, innate immune response, pattern-recognition receptors

## Abstract

Transmissible gastroenteritis virus (TGEV), an enteropathogenic coronavirus (CoV) of porcine, causes lethal watery diarrhea and severe dehydration in piglets and leads to severe economic losses in the swine industry. Unlike most CoVs that antagonize type I interferon (IFN) production, previous studies showed that TGEV infection induces IFN-I production both *in vivo* and *in vitro*. However, the underlying mechanism(s) remain largely unknown. In this study, we found that TGEV infection significantly facilitated IFN-β production as well as activation of the transcription factors IFN regulatory factor 3 (IRF3) and nuclear factor-kappaB (NF-κB) in porcine kidney (PK-15) cells. Screening of TGEV-encoded proteins demonstrated that non-structural protein 14 (nsp14) was the most potent IFN-β inducer and induced IFN-β production mainly by activating NF-κB but not IRF3. Further analysis showed that nsp14 interacted with DDX1, a member of the DExD/H helicase family. Knockdown of DDX1 by specific small interfering RNA (siRNA) significantly decreased nsp14-induced IFN-β production and NF-κB activation. Furthermore, TGEV-induced IFN-β production and IFN-stimulated gene (ISG) expression were decreased in cells transfected with DDX1-specific siRNA, indicating the vital role of DDX1 to TGEV-induced IFN-β responses. In summary, our data revealed a potential coactivator role of host RNA helicase DDX1 to the induction of IFN-β response initiated by TGEV and demonstrated that nsp14 is an important IFN inducer among the TGEV-encoded proteins.

## Introduction

Transmissible gastroenteritis virus (TGEV) is a member of the *Alphacoronavirus* genus within the family *Coronaviridae* in the order *Nidovirales*. TGEV infection mainly causes acute enteric disease characterized by lethal watery diarrhea, severe dehydration, and high mortality in suckling piglets less than 3 weeks old, which has led to severe economic losses in the global swine industry since the first outbreak in 1933 in Ilinois, USA (1, 2). TGEV contains a single-stranded, positive-sense RNA genome of about 28.5 kb (3), including at least nine open reading frames (ORFs). Two slightly overlapping ORFs, ORF1a and ORF1b, located at the 5' two-thirds of the viral genome, encode a replicase complex that is proteolytically processed into 16 non-structural proteins (nsp1 to 16) (4). Structural proteins, nucleocapsid (N) protein, membrane (M) glycoprotein, spike (S) glycoprotein, a small envelope (E) glycoprotein, and accessory proteins 3a, 3b, and 7 are encoded by genes located at the 3′ end (5).

As early as 1981, the presence of high levels of type I interferon (IFN-I) activity in the digestive tract of TGEV-infected newborn piglets was first observed (6). Thereafter, Charley et al. reported that TGEV infection in human or bovine peripheral blood mononuclear cells also induced high IFN-α production (7). Subsequently, Bosworth et al. demonstrated that 2′,5′-oligoadenylate synthetase (OAS), a well-known IFN-stimulated gene (ISG), was increased in TGEV-infected pigs (8). Our previous quantitative proteomics analysis revealed that TGEV infection induced canonical IFN-I signaling through Janus kinase signal transducer and activator of the transcription 1 (JAK-STAT1) pathway, and eight tested ISGs, including IFN-induced protein with tetratricopeptide repeats 1 (IFIT1), IFIT2, IFIT3, OAS1, OAS2, Mx1, Mx2, and ISG15 were upregulated after TGEV infection (9). These early results indicated that TGEV infection activated the IFN-I pathway *in vitro* and *in vivo*. However, the underlying mechanism(s) utilized by TGEV to induce IFN-I, and especially which viral protein(s) contribute to it, remain largely unclear.

The IFN-I response is a well-known innate immune reaction that occurs in response to virus infection and considered as an important bridge between innate and adaptive immunity. Nuclear factor-kappaB (NF-κB) and IFN regulatory factor 3 (IRF3) are two critical transcription factors for the regulation of IFN-I production. Secreted IFN-I then stimulates the JAK-STAT1 signaling pathway to induce the expression of numerous ISGs, which collaborate to regulate the replication of virus (10). Many viruses antagonize IFN responses to benefit their propagation, and some viruses such as human immunodeficiency virus-type 1 (11), vesicular stomatitis virus (VSV) (12), influenza A virus (IAV) (13), encephalomyocarditis virus (14), Reovirus (15), herpes simplex virus 2 (HSV2) (16), respiratory syncytial virus (17), Newcastle disease virus (18), and Sendai virus (SEV) (19) initiate innate immune responses. Different viruses employ different mechanisms to regulate innate immune responses. For example, HSV2 induces IFN-α/β production through toll-like receptor 9 (TLR9), DNA-dependent activator of IFN regulatory factors (DAI), and IFN-inducible 16 (16). For IAV infection, at least TLR3, TLR7, Retinoic acid-inducible gene I (RIG-I), and pyrin domain-containing 3 (NLRP3) are responsible for the detection of IAV and subsequent innate immune responses (13). Elucidating the mechanisms through which viruses regulate innate immune responses will help us understand the interactions between virus and host.

This study sought to identify TGEV-encoded protein(s) involved in the induction of IFN-β production. Our results revealed that TGEV nsp14 was the best inducer of the IFN-β pathway among the TGEV-encoded proteins. Mechanistically, nsp14 activates NF-κB but not IRF3, and it interacts with RNA helicase DDX1, which in turn activates IFN-β production.

## Materials and Methods

### Cells, Viruses, and Antibodies

Porcine kidney (PK-15) cells and HEK-293T cells were cultured in Dulbecco’s modified Eagle’s medium (Invitrogen, Carlsbad, CA, USA) supplemented with 10% fetal bovine serum in a humidified incubator with 37°C/5% CO_2_. TGEV strain WH-1 (GenBank accession no. HQ462571) was propagated and titered in PK-15 cells. Recombinant VSV-expressing green fluorescent protein (VSV-GFP) was generously provided by Prof. Zhigao Bu from the Harbin Veterinary Research Institute, China. Rabbit polyclonal antibodies against p65, IRF3, phosphorylated IRF3 (p-IRF3), and DDX1 were purchased from ABclone (Wuhan, China). Rabbit polyclonal antibody against phosphorylated p65 (p-p65) was purchased from Cell Signaling Technology (Beverly, MA, USA). Anti-β-actin antibody was purchased from Beyotime (Nantong, China). Mouse monoclonal antibodies (MAbs) against hemagglutinin (HA) and Flag were purchased from Medical and Biological Laboratories (MBL, Nagoya, Japan). MAb against TGEV N protein was prepared by our laboratory. Horseradish peroxidase (HRP)-conjugated goat anti-rabbit antibody and HRP-conjugated goat anti-mouse antibody were purchased from MBL. Alexa Fluor 594-conjugated donkey anti-rabbit IgG, Alexa Fluor 594-conjugated donkey anti-mouse IgG, and Alexa Fluor 488-conjugated donkey anti-mouse IgG were obtained from Santa Cruz Biotechnology Inc. (Santa Cruz, CA, USA).

### Plasmids and siRNAs

Expression plasmids of TGEV-encoded proteins used in this study were constructed by RT-PCR amplification from the genomic RNA of TGEV strain WH-1 and cloned into expression vector pCAGGS-HA. The details of primers used for PCR clone are available on request. The p65 gene was derived from human RelA cDNA and cloned into pEGFP-C1 vector. The DDX1 expression plasmid was constructed by RT-PCR amplification from the cDNA of PK-15 cells and cloned into pCMV-tag2B vector. Luciferase reporter plasmids p125-luc (IFN-β-Luc), 4 × PRDIII/I-Luc (referred to as IRF3-Luc), 4 × PRDII-Luc (referred to as NF-κB-Luc), and the internal control plasmid pRL-TK have been described previously (20). Small interfering RNA (siRNA) targeting DDX1 or negative control siRNA (siNC, Invitrogen) were each transfected at a final concentration of 50 nM. The siRNA sequences used here are listed in Table 1.

**Table 1:** Small interfering RNA (siRNA) sequences used in this study.

Gene name siRNA sequence
hDDX1 (sense) GGAGCUUCUGAUAAUUGGAGGUGUU
hDDX1 (anti-sense) AACACCUCCAAUUAUCAGAAGCUCC
pDDX1 (sense) GAAAGACCUUGGUCUGGCAUUUGAA
pDDX1 (anti-sense) UUCAAAUGCCAGACCAAGGUCUUUC

### Transfection and Dual Luciferase Assay

HEK-293T or PK-15 cells were seeded in 24-well plates at a density of 2–4 × 10^5^ cells/well and cultured until the cells reached approximately 70–80% confluence, then transfected with the indicated plasmids or siRNAs using Lipofectamine 2000 (Invitrogen) according to the manufacturer’s protocol. Dual luciferase assays were conducted according to the manufacturer’s instructions (Promega, USA). For each transfection, 0.1 µg of the indicated reporter plasmid along with 0.02 µg of pRL-TK for normalization and 0.2–0.8 µg of various expression plasmids or empty control plasmid were used. Cell extracts were collected at the indicated time points and luciferase activity was measured with a dual-specific luciferase assay kit (Promega). All reporter assays were independently repeated at least three times.

### Co-Immunoprecipitation (CO-IP) and Western Blot Assay

Cells in 6-well plates were harvested by the addition of 150 µl of lysis buffer (4% sodium dodecyl sulfate, 3% dithiothreitol, 0.065 mM Tris–HCl [pH 6.8], 30% glycerine). Equal amounts of samples were subjected to sodium dodecyl sulfate polyacrylamide gel electrophoresis (SDS-PAGE) and electroblotted onto a polyvinylidene difluoride membrane (Bio-Rad). Protein expression was analyzed by immunoblotting with the indicated antibodies. Expression of β-actin was detected to demonstrate equal protein sample loading. For CO-IP experiments, HEK-293T cells in 100-mm dishes were transiently transfected with the indicated plasmids. Cells were washed with phosphate-buffered saline (PBS) and lysed for 30 min at 4°C in 1 ml lysis buffer [50 mM Tris-HCl (pH 7.5), 150 mM NaCl, 1% NP-40, 20 nM phenylmethylsulfonyl fluoride], and protein concentration was measured and adjusted. For each immunoprecipitation, 500 µg of cell lysate protein was incubated with 2 µg of indicated antibody and 25 µl of protein A/G-agarose (Beyotime) overnight at 4°C. After three washes with 1 ml lysis buffer, precipitates were subjected to 10% SDS-PAGE and subsequently analyzed with immunoblot analysis using the indicated antibodies.

### Indirect Immunofluorescence Assay

Porcine kidney (PK)-15 cells were fixed with 4% paraformaldehyde for 15 min followed by permeabilization with pre-cooled methanol for 10 min, blocking with 5% bovine serum albumin for 45 min, incubated with the indicated primary antibodies for 1 h, followed by staining with specific Alexa Fluor-conjugated secondary antibodies for 1 h. The cells were subsequently stained with 4',6-diamidino-2-phenylindole (Beyotime) for 15 min. After washing with PBS, fluorescent images were obtained using an Olympus FV10 laser scanning confocal microscope (Olympus, Japan).

### RNA Extraction and Real-time RT-qPCR

Total RNAs were extracted using TRIzol reagent (Invitrogen). Real-time RT-qPCR was performed using SYBR Green Real-Time PCR Master Mix (Toyobo Biologics, Osaka, Japan) in the ABI PRISM 7000 sequence detection system (Applied Biosystems). Individual transcripts in each sample were assayed three times. The fold change in gene expression relative to normal was calculated using the delta-delta cycles to threshold (ΔΔCT) method. Primers (Table 2) were designed using Primer Express software (version 3.0; Applied Biosystems, Carlsbad, CA, USA).

**Table 2:** Primers used for real-time RT-qPCR.

Primer names	Sequence (5′–3′)
pIFIT1(ISG56)-qF	AAATGAATGAAGCCCTGGAGTATT
pIFIT1(ISG56)-qR	AGGGATCAAGTCCCTACAGATTTT
pIFIT2(ISG54)-qF	CTGGCAAAGAGCCCTAAGGA
pIFIT2(ISG54)-qR	CTCAGAGGGTCAATGGAATTCC
pIFIT3(ISG60)-qF	GAAACCCGACAACCCAGAATT
pIFIT3(ISG60)-qR	GCTGGTTTGCCATTCAGAAAG
pIL6-qF	CTGCTTCTGGTGATGGCTACTG
pIL6-qR	GGCATCACCTTTGGCATCTT
pIL8-qF	AGTTTTCCTGCTTTCTGCAGCT
pIL8-qR	TGGCATCGAAGTTCTGCACT
pIFN-β-qF	GCTAACAAGTGCATCCTCCAAA
pIFN-β-qR	AGCACATCATAGCTCATGGAAAGA
pGAPDH-qF	ACATGGCCTCCAAGGAGTAAGA
pGAPDH-qR	GATCGAGTTGGGGCTGTGACT
hIFN-β-qF	TCTTTCCATGAGCTACAACTTGCT
hIFN-β-qR	GCAGTATTCAAGCCTCCCATTC
hGAPDH-qF	TCATGACCACAGTCCATGCC
hGAPDH-qR	GGATGACCTTGCCCACAGCC

### Statistical Analysis

All experiments were performed at least three times with reproducible results. Data are presented as the mean ± SD. Statistical analysis was performed using one-way ANOVA without interaction terms followed by Dunnett’s for multiple comparisons.

### Ethical Statement

All animal experiments were approved by the Hubei Administrative Committee for Laboratory Animals (permission number 00024534) and complied with the guidelines of Hubei laboratory animal welfare and ethics of Hubei Administrative Committee of Laboratory Animals.

## Results

### TGEV Infection Stimulates IFN-β Production in PK-15 Cells

**Figure 1:**
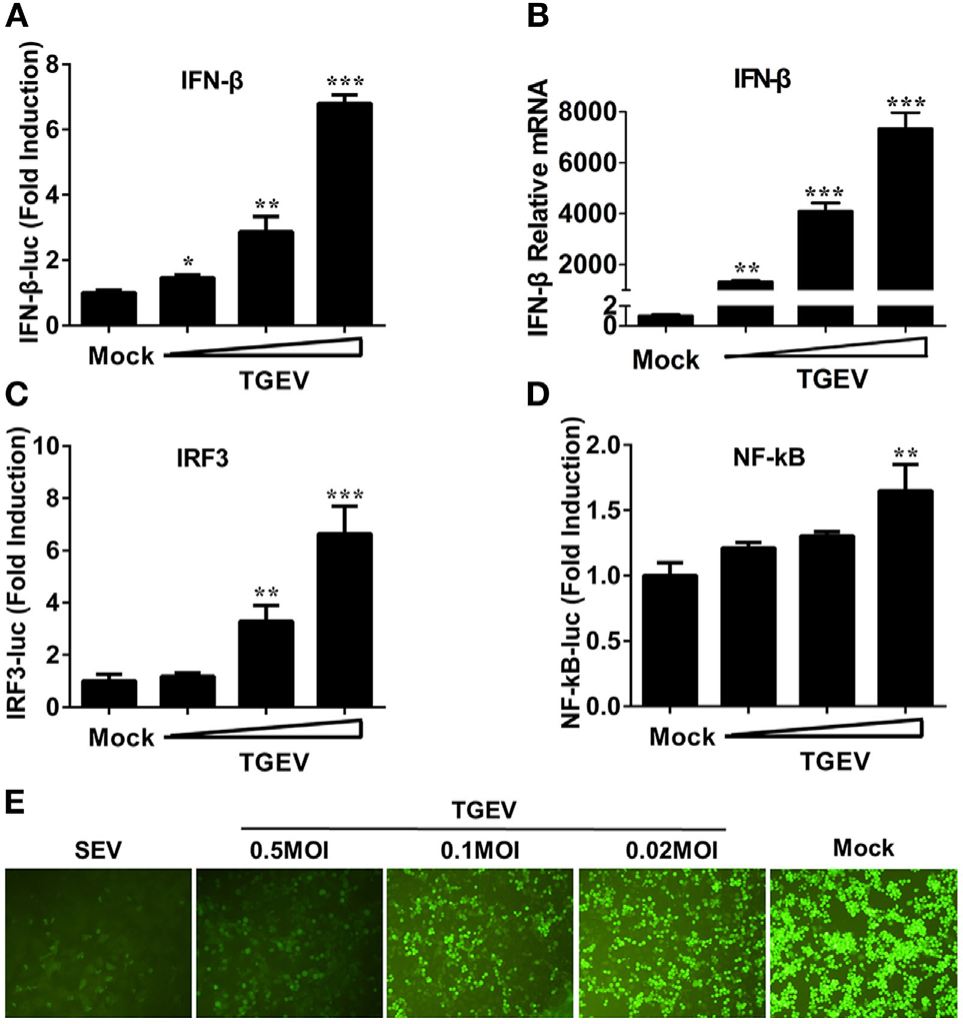
Transmissible gastroenteritis virus (TGEV) dose-dependently triggers activation of the IFN-β signal pathway and suppresses the replication of VSV. PK-15 cells were cotransfected with pRL-TK and IFN-β-Luc **(A)**, IRF3-Luc **(C)**, or NF-κB-Luc **(D)**, followed by infection with increasing doses of TGEV [multiplicity of infection (MOI) = 0.02, 0.1, or 0.5] at 12 h post-transfection. The cells lysates were collected for dual luciferase assays at 24 hpi. **(B)** PK-15 cells were infected with TGEV as described in panel A and harvested at 24 hpi. Cell RNAs were extracted for RT-qPCR to examine the mRNA expression levels of IFN-β. The mRNA expression levels were normalized to porcine GAPDH transcripts. Values are the mean ± SD of three independent tests. *P < 0.05, **P < 0.01, or ***P < 0.001 compared with the mock infection group. **(E)** PK-15 cells were infected or mock infected with TGEV (MOI = 0.02, 0.1, 0.5). The supernatants were collected for UV-irradiation and then transferred onto fresh PK-15 cells, followed by infection with vesicular stomatitis virus expressing green fluorescent protein (VSV-GFP) at 24 h later. Cells were observed with fluorescence microscope at 16 hpi. The supernatants from SEV-treated cells served as a positive control.

Previous studies demonstrated that TGEV infection potently induced IFN-α (6, 21, 22), as well as IFN-β in IPEC-J2 and swine testicular (ST) cells (23–25). However, whether TGEV infection induces IFN-β in PK-15 cells remains unknown. To explore the effect of TGEV on IFN-β, dual luciferase assays were performed. PK-15 cells were transfected with IFN-β-Luc and pRL-TK. After 12 h, cells were mock infected or infected with TGEV strain WH-1 at a multiplicity of infection (MOI) of 0.02, 0.1, or 0.5. As shown in Figure 1A, TGEV infection significantly induced the activation of the IFN-β promoter in a dose-dependent manner. Moreover, IFN-β mRNA expression levels were upregulated in TGEV-infected cells dose-dependently, which further confirmed IFN-β production by TGEV infection (Figure 1B).

The induction of IFN-I is reliant on the co-regulation of transcription factors IRF3 and NF-κB. To investigate the potential mechanism(s) involved in the IFN-β production by TGEV infection, the effect of TGEV on IRF3 and NF-κB promoters were also tested. As displayed in Figures 1C,D, TGEV infection also upregulated IRF3 and NF-κB promoter activity dose-dependently, indicating that IRF3 and NF-κB are involved in the IFN-β production by TGEV infection.

Because of the high sensitivity of VSV-GFP to IFN, VSV-GFP expression is commonly monitored for IFN detection. To further evaluate the IFN-β response in TGEV-infected cells, a VSV-GFP-based IFN detection assay was performed. PK-15 cells were infected or mock infected with SEV or TGEV (MOI = 0.02, 0.1, 0.5). Then, cell supernatants were collected, UV-irradiated, and then transferred onto fresh PK-15 cells. After 24 h, cells were infected with VSV-GFP and observed under a fluorescence microscope at 16 hpi. As a positive control, supernatants collected from SEV-treated cells suppressed VSV-GFP replication prominently compared with the negative control group (mock). In accordance with the results of dual luciferase assays described above, TGEV infection limited the replication of VSV-GFP in a dose-dependent manner (Figure 1E). These results suggested that TGEV infection increased IFN-β production.

### TGEV nsp14 Induces IFN-β Activation

**Figure 2:**
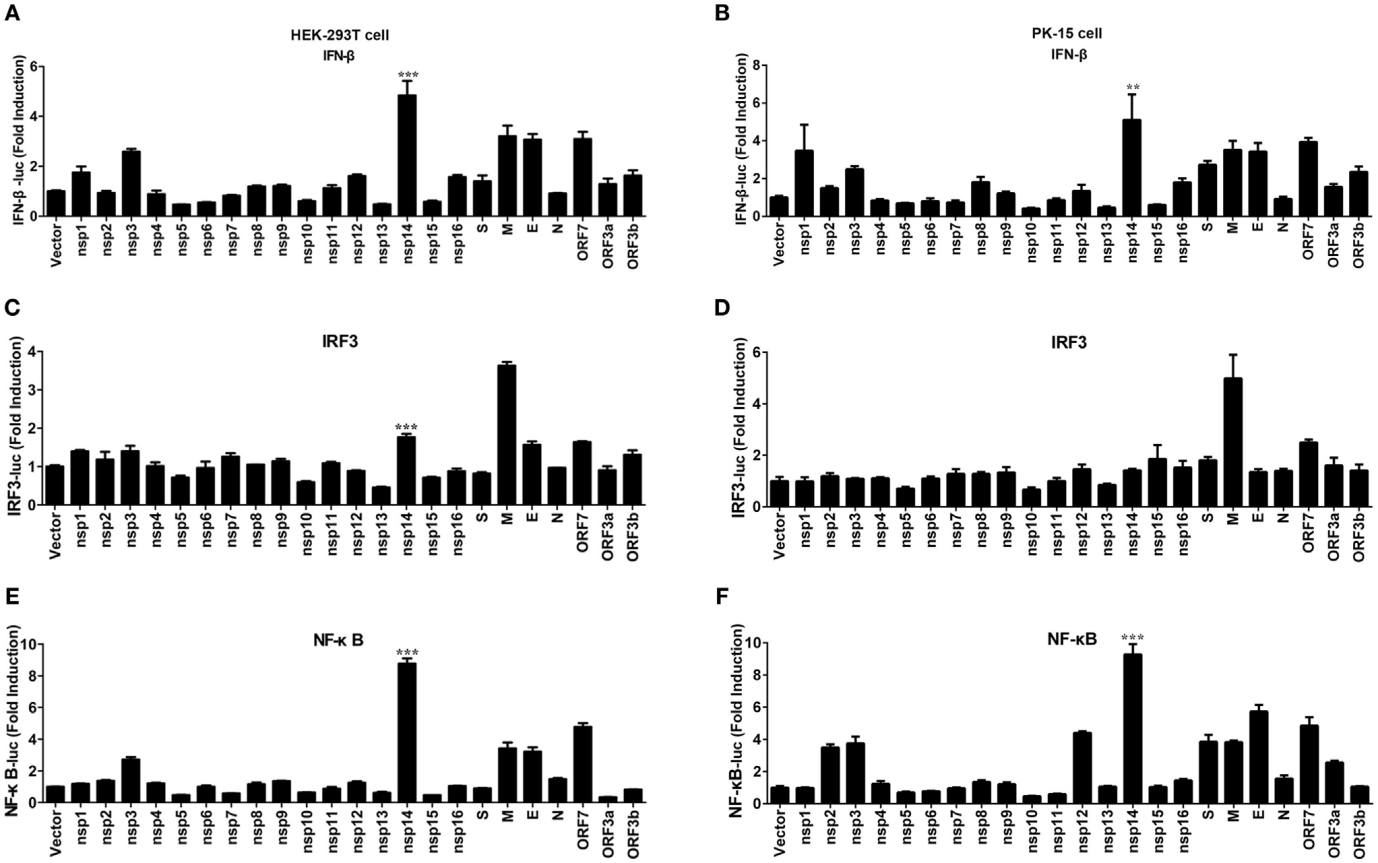
Effect of transmissible gastroenteritis virus (TGEV) proteins on IFN-β, IRF3, and NF-κB promoter. HEK-293T cells were cotransfected with pRL-TK and IFN-β-Luc **(A)**, IRF3-Luc **(C)**, or NF-κB-Luc **(E)** together with each expression plasmid of TGEV proteins. Also, PK-15 cells were cotransfected with pRL-TK and IFN-β-Luc **(B)**, IRF3-Luc **(D)**, or NF-κB-Luc **(F)** together with each expression plasmid of TGEV proteins. The cell lysates were harvested for dual luciferase assays at 30 h post-transfection. Values are the mean ± SD of three independent tests. ***P < 0.001 or **P < 0.01 compared with empty vector group.

Transmissible gastroenteritis virus encodes 16 non-structural proteins (nsp1-16), four structural proteins (N, M, S, E), and three accessory proteins (ORF7, ORF3a, ORF3b). To identify the key viral protein(s) involved in IFN-β induction, an IFN promoter-reporter system was employed to screen TGEV-encoded proteins for their relative capacities to activate the IFN-β promoter. HEK-293T cells were cotransfected with IFN-β-Luc, pRL-TK, and different TGEV protein expression vectors. As shown in Figure 2A, nsp14 was the most significant inducer of IFN-β production. Furthermore, similar to SARS-(coronavirus) CoV (26), TGEV M glycoprotein also potently mediated IFN-β induction. Because NF-κB and IRF3 are necessary transcription factors for IFN-β production, we examined the effects of all TGEV-encoded proteins on IRF3 and NF-κB promoter activity. Interestingly, nsp14 upregulated an approximately 8.8-fold change in NF-κB promoter activity, but only an approximately 1.8-fold change in IRF3 promoter activity (Figures 2C,E). M glycoprotein induced a higher fold change in IRF3, but a lower fold change in NF-κB compared with nsp14. Because M glycoprotein has been investigated previously (27), we focused on nsp14.

To confirm the ability of nsp14 to induce IFN-β production, large-scale screen experiment was also conducted in PK-15 cells, a permissive cell line of TGEV infection. As shown in Figures 2B,D,F, nsp14 was also a potent IFN inducer that mainly induced activation of NF-κB but not IRF3 promoter in PK-15 cells.

### Nsp14 Induces Phosphorylation and Nuclear Translocation of p65

**Figure 3:**
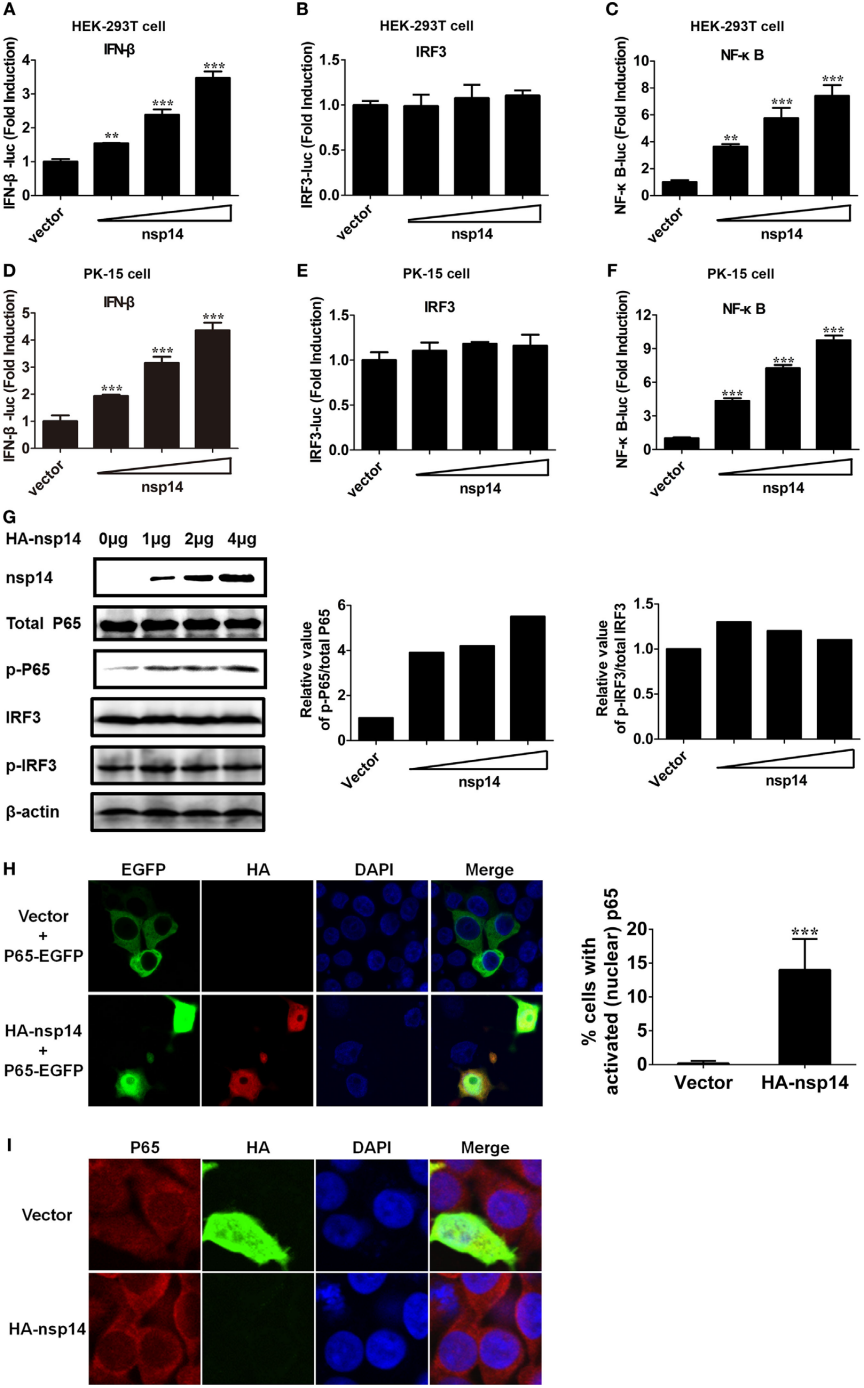
Transmissible gastroenteritis virus nsp14 triggers IFN-β and NF-κB activation. HEK-293T cells were cotransfected with pRL-TK plasmid and IFN-β-Luc **(A)**, IRF3-Luc **(B)**, or NF-κB-Luc **(C)** luciferase reporter plasmids together with increasing doses (0.2, 0.4, 0.8 µg) of pCAGGS-HA-nsp14 or empty vector. Also, PK-15 cells were cotransfected with pRL-TK plasmid and IFN-β-Luc **(D)**, IRF3-Luc **(E)**, or NF-κB-Luc **(F)** luciferase reporter plasmids together with increasing doses (0.2, 0.4, 0.8 µg) of pCAGGS-HA-nsp14 or empty vector. The cells lysates were harvested for dual luciferase assays at 30 h post-transfection. Values are the mean ± SD of three independent tests. **P < 0.01 or ***P < 0.001 compared with empty vector group. **(G)** HEK-293T cells were transfected with increasing quantities (1, 2, 4 µg) of pCAGGS-HA-nsp14 or empty vector for 30 h, and then subjected to immunoblotting with antibodies specific for endogenous IRF3, phosphorylated IRF3 (p-IRF3), p65, or p-p65. Anti-HA mouse antibody was used to confirm the expression of nsp14. β-actin expression was used as a loading control. The ratio of phosphorylated/total p65 and phosphorylated/total IRF3 was analyzed using ImageJ Software. **(H)** PK-15 cells were cotransfected with plasmids encoding HA-tagged nsp14 protein or empty vector together with plasmids encoding EGFP-tagged p65 protein. Then, the cells were fixed and immunostained with anti-HA monoclonal antibodies (mAbs) to observe the nuclear translocation of overexpressed p65 using confocal microscopy. **(I)** HEK-293T cells were transfected with plasmids encoding HA-tagged nsp14 protein or empty vector. Then, the cells were fixed and immunostained with anti-p65 antibody to observe the nuclear translocation of endogenous p65 using confocal microscopy.

To confirm the above large-scale screen results, increasing doses (0.2, 0.4, 0.8 µg) of pCAGGS-HA-nsp14 and IFN-β-Luc, IRF3-Luc or NF-κB-Luc together with pRL-TK were cotransfected into HEK-293T cells. In line with the results in Figure 2, nsp14 enhanced the activation of IFN-β and NF-κB in a dose-dependent manner (Figures 3A–C). Interestingly, nsp14 induced the activation of NF-κB to a greater extent than that of IRF3, indicating NF-κB has a fundamental role in nsp14-induced IFN-β activation. Similar results were also obtained in PK-15 cells (Figures 3D–F).

NF-κB and IRF3 activation are characterized by the phosphorylation and subsequent translocation of p65 (NF-κB subunit) or IRF3 to the nucleus, respectively (28–30). Next, we investigated the role of nsp14 in the phosphorylation of p65 and IRF3. HEK-293T cells were transfected with increasing amounts of pCAGGS-HA-nsp14, and cell lysates were examined for the expression levels of p-p65 or p-IRF3 and total p65 or IRF3 at 30 h post-transfection. As shown in Figure 3G, nsp14 overexpression had no significant effect on the amount of p65, IRF3, and p-IRF3; however, markedly increased p65 phosphorylation levels, indicating the activation of NF-κB, rather than IRF3 is associated with nsp14-induced IFN-β production. Therefore, the subcellular location of p65 was further investigated after nsp14 overexpression. As shown in Figures 3H,I, ectopic expression of nsp14 resulted in the nuclear translocation of overexpressed p65 in PK-15 cells (Figure 3H) and endogenous p65 in HEK-293T cells (Figure 3I).

### Nsp14 Interacts with DDX1

**Figure 4:**
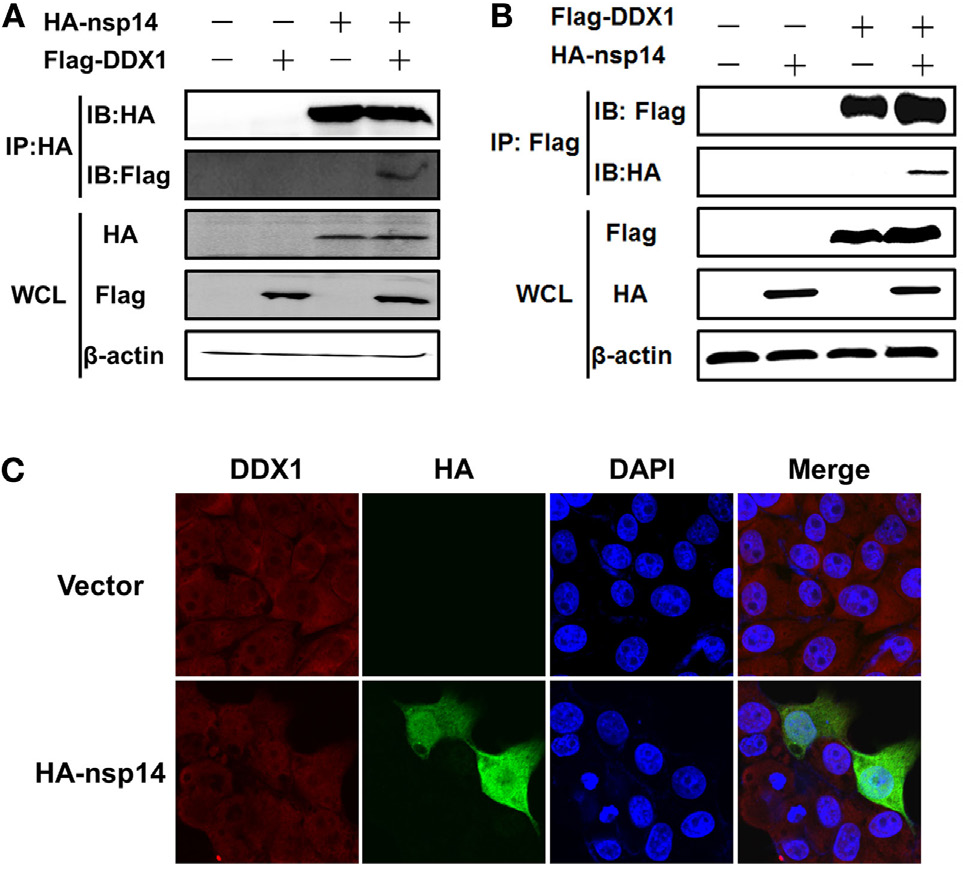
Interaction between transmissible gastroenteritis virus nsp14 and DDX1. **(A,B)** HEK-293T cells were cotransfected with pCAGGS-HA-nsp14 and/or pCMV-Tag2B-DDX1 and/or empty vector(s). Cell lysates were treated with RNase and co-immunoprecipitated with anti-HA **(A)** or anti-Flag **(B)** mAbs followed by immunoblot of nsp14 and DDX1 using anti-HA and -Flag mAbs to assess the interaction between nsp14 and DDX1 protein. **(C)** Colocalization of DDX1 with nsp14. PK-15 cells were transfected with pCAGGS-HA-nsp14 or empty vector. Then, the cells were fixed at 30 h post-transfection and immunostained with anti-HA mouse antibody and anti-DDX1 rabbit antibody to detect the colocalization of DDX1 with nsp14.

Early studies suggested that nsp14 of CoV IBV and SARS-CoV interacts with host protein DDX1 (31). Therefore, we investigated whether TGEV nsp14 also interacts with DDX1 by CO-IP. HEK-293T cells were cotransfected with pCAGGS-HA-nsp14 and pCMV-Tag2B-DDX1. Because DDX1 is a DExD/H-box helicase and is associated with RNA metabolism, the lysates were treated with RNase to avoid the effect of RNA on the CO-IP experiment. As shown in Figure 4A, Flag-tagged DDX1 was coprecipitated by HA-tagged nsp14, indicating the interaction between nsp14 and DDX1. Reversed IP with Flag antibody further confirmed this interaction (Figure 4B).

To test whether the colocalization of nsp14 and DDX1 occurs, a prerequisite for the interaction, PK-15 cells were transfected with pCAGGS-HA-nsp14 or empty vector and fixed at 30 h post-transfection. HA-tagged nsp14 protein was detected with mouse anti-HA antibody, and DDX1 was detected with rabbit anti-DDX1 antibody. The results revealed that nsp14 was colocalized with DDX1 and distributed both in the cytoplasm and nucleus (Figure 4C), which further confirmed the interaction between TGEV nsp14 and DDX1.

### DDX1 Is Involved in nsp14-Induced IFN-β Production

**Figure 5:**
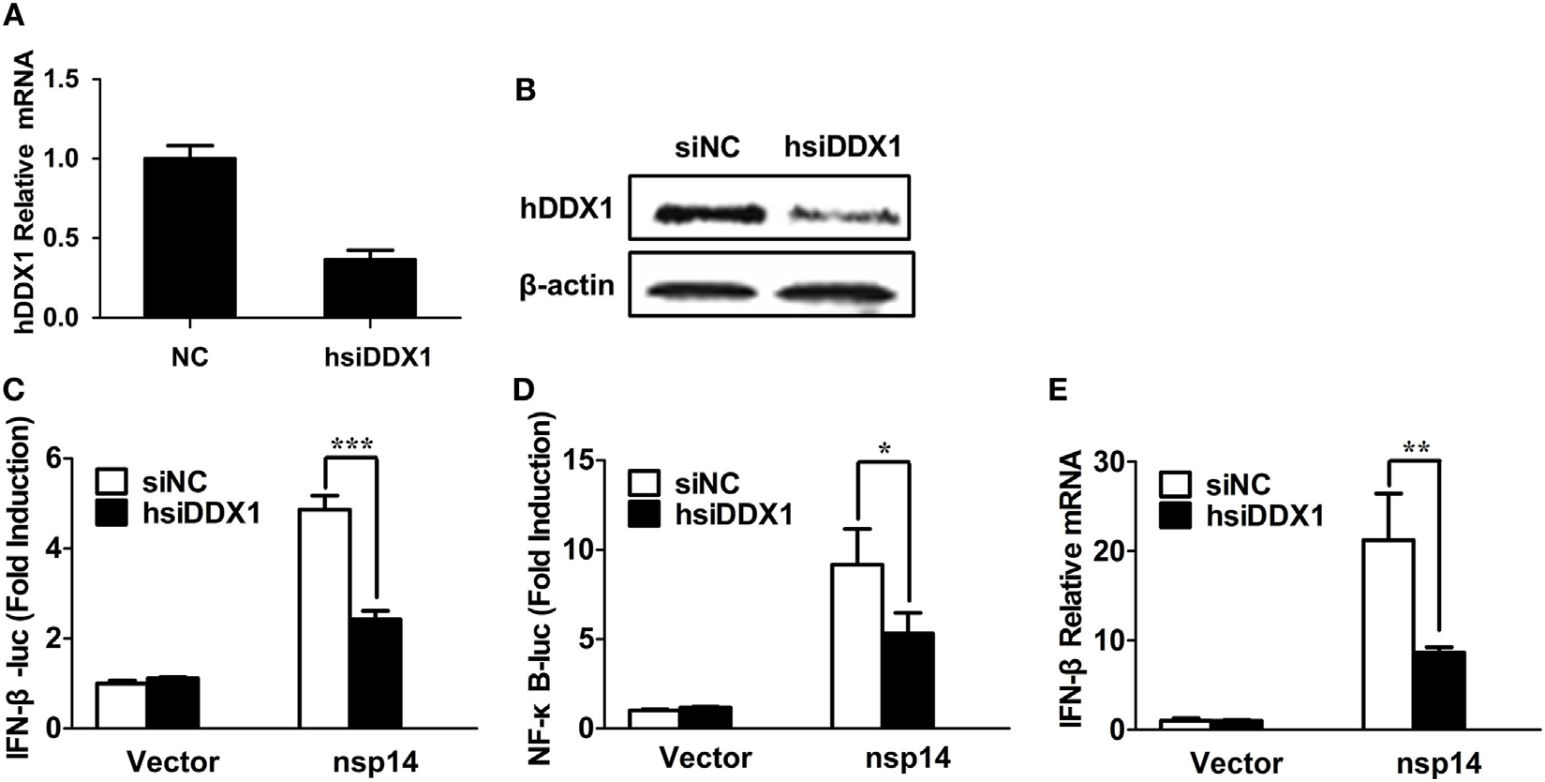
Silencing DDX1 decreases nsp14-stimulated IFN-β and NF-κB activation. **(A,B)** Assessment of the silencing efficiency of hsiDDX1. HEK-293T cells were transfected with 50 nM/well of hsiDDX1 or siNC for 36 h each. The expression levels of human DDX1 were determined by RT-qPCR **(A)** and western blot assay **(B)**. **(C,D)** HEK-293T cells were cotransfected with psiDDX1 or siNC, together with pCAGGS-HA-nsp14 or empty vector, pRL-TK, IFN-β-Luc **(C)**, or NF-κB-Luc **(D)**. Dual luciferase assays were performed at 30 h post-transfection. **(E)** HEK-293T cells were cotransfected with psiDDX1 or siNC, together with pCAGGS-HA-nsp14 or empty vector and harvested at 30 h post-transfection. Cell RNAs were extracted for RT-qPCR to examine the mRNA expression levels of IFN-β. The mRNA expression levels were normalized to human GAPDH transcripts. All data represent the mean ± SD of three independent experiments. *P < 0.05 or ***P < 0.001 compared with the siNC group.

Because nsp14 activates IFN-β and interacts with DDX1, we investigated whether DDX1 is involved in nsp14-induced IFN-β production. Synthesized siRNA targeting human DDX1 (hsiDDX1), which efficiently decreases the expression of endogenous DDX1 mRNA (Figure 5A) and protein (Figure 5B), was selected. Next, HEK-293T cells were transfected with hsiDDX1 or siNC, followed by co-transfection of IFN-β-luc or NF-κB-Luc, pRL-TK, along with pCAGGS-HA-nsp14 or empty vector. The results revealed that knockdown of DDX1 significantly decreased nsp14-induced promoter activity of IFN-β (Figure 5C) and NF-κB (Figure 5D). Moreover, mRNA expression levels of nsp14-induced IFN-β were downregulated by DDX1 deficiency (Figure 5E), suggesting the involvement of DDX1 in IFN-β induction by nsp14.

### DDX1 Is Involved in TGEV-Induced IFN-β Activation and ISG Expression

**Figure 6:**
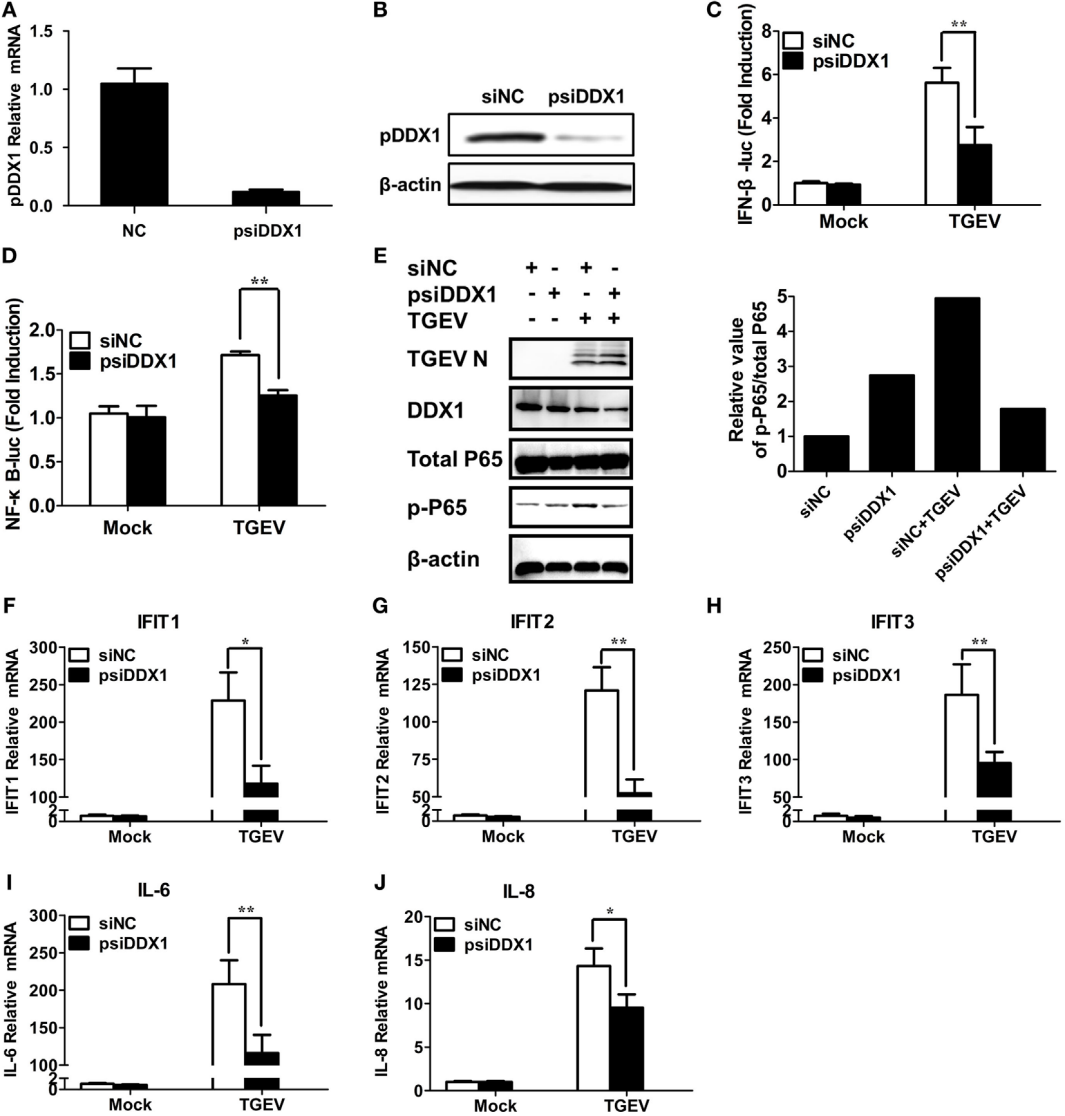
Involvement of DDX1 in the antiviral response triggered by transmissible gastroenteritis virus (TGEV). **(A,B)** Assessment of the silencing efficiency of psiDDX1. PK-15 cells were transfected with 50 nM/well of psiDDX1 or siNC for 36 h each. The expression levels of porcine DDX1 were determined by RT-qPCR **(A)** and western blot assay **(B)**. **(C,D)** PK-15 cells were cotransfected with psiDDX1 or siNC, along with pRL-TK, IFN-β-Luc **(C)**, or NF-κB-Luc **(D)**. At 12 h post-transfection, cells were mock infected or infected with TGEV [multiplicity of infection (MOI) = 0.5]. Dual luciferase assays were performed at 24 hpi. **(E)** PK-15 cells were transfected with psiDDX1 or siNC, and 12 h later, cells were mock infected or infected with TGEV (MOI = 0.5) for 24 h. Then, the cells were collected for western blot assay with specific antibodies against p65, p-p65, DDX1, or TGEV N protein, using β-actin expression as a loading control. The ratio of phosphorylated/total p65 was analyzed using ImageJ Software. **(F–J)** PK-15 cells were treated as described for **(E)** and collected at 24 hpi. Cell RNAs were extracted for RT-qPCR to examine the mRNA expression levels of IFIT1 **(F)**, IFIT2 **(G)**, IFIT3 **(H)**, IL-6 **(I)**, and IL-8 **(J)**. The mRNA expression levels were normalized to porcine GAPDH transcripts. Values are the mean ± SD of three independent tests. *P < 0.05 or **P < 0.01 compared with the siNC group.

To determine whether DDX1 is involved in TGEV-induced IFN-β activation, we designed three pairs of siRNAs targeting porcine DDX1 (psiDDX1) and selected one with the best knockdown efficiency as demonstrated by RT-qPCR (Figure 6A) and western blot assay (Figure 6B), for subsequent experiments. PK-15 cells were cotransfected with IFN-β-Luc or NF-κB-luc, pRL-TK, together with psiDDX1 or siNC. At 12 h post-transfection, cells were infected with TGEV for 24 h, followed by dual luciferase assay. DDX1 depletion had no effect on the basal activity of IFN-β and NF-κB promoter, but significantly decreased TGEV-induced activation of IFN-β and NF-κB (Figures 6C,D). We also detected the expression level of p-p65 in TGEV-infected PK-15 cells when DDX1 was silenced. As shown in Figure 6E, knockdown of DDX1 reduced TGEV-induced p65 phosphorylation. These results suggested that DDX1 is associated with TGEV-induced IFN-β and NF-κB activation.

Interferon-I initiates a series of signaling cascades through the JAK/STAT pathway, resulting in the expression of numerous ISGs (32). Furthermore, NF-κB activation plays a pivotal role in regulating the transcription and expression of many proinflammatory cytokines. Because DDX1 is involved in TGEV-induced IFN-β and NF-κB activation, theoretically, it should have an impact on TGEV-induced ISG and pro-inflammatory cytokine expression. The expression levels of some ISGs (IFIT1, IFIT2, IFIT3) and proinflammatory cytokines (IL-6, IL-8) in DDX1-knockdown cells were analyzed after TGEV infection. As expected, DDX1 depletion inhibited the expression of IFIT1, IFIT2, IFIT3, as well as IL-6 and IL-8 to some degree, compared with that in cells transfected with siNC (Figures 6F–J).

## Discussion

The innate immune response characterized by the synthesis of IFN and proinflammatory cytokines is the first line of antiviral defense. Multiple studies have reported the involvement of CoVs in the regulation of innate immune responses. The majority of CoVs decreased dsRNA-mediated IFN-β production, including porcine epidemic diarrhea virus (PEDV) (33), severe acute respiratory syndrome coronavirus (SARS-CoV) (34), and infectious bronchitis virus (IBV) (35). Interestingly, mouse hepatitis virus (MHV)-induced IFN-α/β and established an antiviral state in plasmacytoid dendritic cells and macrophages, but failed to produce IFN in neurons, astrocytes, and hepatocytes (36, 37), indicating that MHV induction of IFN-α/β is cell type dependent. A previous study showed that TGEV-induced IFN-α secretion *in vitro* and *in vivo* (38). Here, we found that TGEV infection increased the production of IFN-β in a dose-dependent manner in PK-15 cells, in line with previously reported data (25).

The significant roles of many CoV proteins in the regulation of innate immune response have been identified. For example, nsp1, nsp7, nsp15, Papain-like protease (PLpro), ORF3b, ORF6, ORF9b, and N protein of SARS-CoV have been identified as IFN antagonists *via* various mechanisms (39). PEDV-encoded PLpro, N protein, and 3C-like protease nsp5 inhibit IFN production (40). The current study used an IFN-β promoter-reporter system and found that several TGEV-encoded proteins, particularly nsp14 and M glycoprotein, induced the activation of the IFN-β and/or NF-κB pathway, which also regulate the IFN-I response mediated by other CoVs. Lui et al. reported that the MERS-CoV M glycoprotein is an IFN antagonist because it specifically inhibited IRF3 activation but not NF-κB signaling (41). Conversely, our data revealed that the TGEV M glycoprotein was a potential IFN inducer that mainly facilitated IRF3 activation but not NF-κB signaling. Laude et al. also revealed the direct role of M glycoprotein in the induction of IFN-α by TGEV infection (27). Similarly, M glycoprotein is vital to the induction of IFN by MHV infection in LMR cells (42). However, the effects of SARS-CoV M glycoprotein on IFN-I responses are controversial. Wang et al. reported that the SARS-CoV M glycoprotein induced IFN-I production *via* a toll-like receptor-related TRAF3-independent mechanism (26). In contrast, other researchers reported the opposite conclusion that the SARS-CoV M glycoprotein suppressed NF-κB activation (43) and inhibited IFN-I production by preventing formation of the TRAF3–TANK–TBK1/IKKε complex (44). Wang et al. suggested that these discrepancies may be attributed to the amino acid substitution in the M glycoproteins of different SARS-CoV strains (45). Interestingly, Becares et al. found that rTGEV-zinc finger 1 (ZF-C) (a recombinant TGEV engineered mutation within ZF-C domain of nsp14) infection resulted in the significant inhibition of antiviral responses at different stages compared with rTGEV-WT infection, including the expression of IFN-β, tumor necrosis factor, and ISGs (25). This indicated the potential effect of TGEV nsp14 on IFN-β induction, which is in line with our data and further confirms our conclusion using a reverse genetic system.

Although nsp14 was identified as a key IFN-β activator among TGEV-encoded proteins, it remains unclear which PRR(s) is involved in its detection and induction of IFN-β production. Indeed, viral proteins, similar to viral nucleic acids or replication intermediates, can in some cases also function as PAMPs, specifically recognized by certain host PRRs, such as TLR2 and TLR4, to modulate the IFN responses during viral infection (46, 47). For example, the M glycoprotein of SARS-CoV has been reported to function as a novel cytosolic PAMP to promote IFN-β production by activating a non-canonical TLR signaling cascade (26). In addition to the four major PRR groups reported previously, including TLRs, RIG-I-like receptors, NOD-like receptors and cytoplasmic DNA receptors (48), multiple DExD/H-box helicases, such as DDX1, DDX3, DDX9, and DDX41, were reported recently to act as PRRs and sense viral PAMPs to activate the NF-κB signaling pathway and induce IFN-β production (49–53). Previous study revealed that DDX1, DDX21, and DHX36 form a complex with the adaptor molecule TRIF to sense dsRNA in dendritic cells (53). In this paper, DDX1 interacted with TGEV nsp14 in a RNA-independent manner and enhanced both TGEV- and nsp14-induced activation of IFN-β responses. However, the direct interactions between nsp14 and DDX21 or DHX36 were not observed. We speculated that nsp14 may be sensed by DDX1/DDX21/DHX36 complex by interacting with DDX1. These results suggest that nsp14 may be recognized as PAMP by DDX1, which triggers an antiviral response. Further studies are required to investigate this in more detail.

DDX1 is a DExD/H helicase family member composed of the DEAD-box and related DEAH, DexH, and DexD protein family and is involved in multiple cellular processes of RNA metabolism (54, 55). Besides these traditional roles, it appears that multiple proteins of the DexD/H-box helicase family are associated with viral components and/or have alternative effects on viral propagation (56–58). For instance, DDX3 shows antiviral functions against vaccinia virus, DENV, and HBV (59–61), but is of benefit for HCV and HIV infection (62, 63). In addition, DDX1 interacts with the nsp3 of Venezuelan equine encephalitis virus and enhances viral multiplication (64). The interaction of DDX1 with human immunodeficiency virus type 1 Rev protein is involved in the regulation of virus replication (65). In the present study, the knockdown and ectopic expression of DDX1 demonstrated that DDX1 had antiviral activity against TGEV replication (data not shown). Interestingly, earlier studies also showed an interaction between DDX1 with coronavirus (IBV and SARS-CoV) nsp14, and in contrast to TGEV, this interaction might enhance the replication of IBV (31). This difference suggests that DDX1 is not likely to be a general target against CoV infection.

Furthermore, it should be noted that the effect of DDX1 on progeny TGEV production was moderate (data not shown). Difference from other CoVs, such as SARS-CoV and MERS-CoV which antagonize IFN-I, TGEV infection induces IFN-I production, and a most recent paper showed that poly(I:C)-induced IFN-I responses could only inhibit TGEV replication in the early infection stage, but failed in the late infection stage (23). They also demonstrated that the activation of IFN-I responses by TGEV infection cannot inhibit viral replication. Our results are consistent with the conclusion proposed by Zhu and colleagues. In addition, it is surprising that the expression levels of IFN-β are paralleled with the increase of viral RNA during TGEV infection. These may explain why the effect of DDX1 on TGEV replication was moderate accompany with its significant role in IFN-β induction by TGEV. However, more studies are required to investigate the complex interaction between TGEV and IFNs.

In conclusion, our data demonstrate that TGEV infection induces IFN-β production and nsp14 is the most significant IFN-β inducer among the TGEV-encoded proteins. Nsp14 interacts with cellular DExD/H helicase DDX1 to activate IFN-β in a NF-κB dependent manner, and DDX1 is associated with TGEV-induced IFN-β production, revealing a potential coactivator role of host RNA helicase DDX1 on virus and viral protein induced innate immune responses.

## Ethics Statement

All animal experiments were approved by the Hubei Administrative Committee for Laboratory Animals (permission number 00024534) and complied with the guidelines of Hubei laboratory animal welfare and ethics of Hubei Administrative Committee of Laboratory Animals.

## Author Contributions

YZ, WW, and LF designed research; YZ, WW, and LX performed research; YZ, YF, DW, and SX analyzed data; YZ and LF wrote the first draft of the manuscript. HC, QK, ZH, and XW contributed to modify the manuscript. All the authors read and approved the manuscript.

## Conflict of Interest Statement

The authors declare that the research was conducted in the absence of any commercial or financial relationships that could be construed as a potential conflict of interest.
